# Patient-reported side effects immediately after chiropractic scoliosis treatment: a cross-sectional survey utilizing a practice-based research network

**DOI:** 10.1186/s13013-015-0053-8

**Published:** 2015-10-05

**Authors:** A. Joshua Woggon, Dennis A. Woggon

**Affiliations:** CLEAR Scoliosis Institute, 437 North 33rd Ave, Saint Cloud, MN 56303 USA

## Abstract

**Background:**

Concern exists regarding the potential for chiropractic treatment to cause adverse effects in individuals with scoliosis. The aim of this paper is to present the self-reported responses of 189 scoliosis patients over 3198 unique visits, collected over one calendar year from nine chiropractic clinics, regarding how they felt and the side effects they experienced immediately after chiropractic treatment.

**Methods:**

Thirty six private chiropractic clinics specializing in the treatment of scoliosis were asked to participate in a prospective study regarding the side-effects of the chiropractic treatment of scoliosis; 9 agreed to participate. A response form was provided to each scoliosis patient at the end of their clinic visit, and consisted of two questions: “How do you feel after your treatment today?” and “Did you experience any side-effects as a result of your treatment today?”

**Results:**

One hundred eighty nine informed consent forms were collected and 3198 response forms were collected, suggesting an average of 17 visits per patient. Patients reported feeling worse post-treatment after 5.0 % of the visits. The incidence of side-effects was 29.7 %. Muscle soreness accounted for 35.2 % of all side effects. 99.9 % of all side effects were classified as mild. Six moderate side-effects (sprains/strains) were reported out of 3,198 visits. There were no reported cases of severe side effects.

**Conclusion:**

Mild side effects were common, although the frequency was slightly lower than the average for chiropractic interventions. The rate of moderate side effects reported was one per 533 visits involving the care of 189 scoliosis patients surveyed from 9 chiropractic offices over a timeframe of one calendar year. No serious adverse events occurred that required medical attention, hospital stays, or surgical intervention. Based upon this preliminary data, side effects reported by scoliosis patients immediately after chiropractic treatment appear to be relatively common but generally benign.

## Background

Scoliosis is a three-dimensional deformity that results in changes to the structure and function of the spine as well as the para-spinal soft tissue structures, and may be linked to imbalances or dysfunctions in the proprioceptive and vestibular systems. It is commonly diagnosed in adolescence, but can be found in adults as well.

Conventional treatments for scoliosis include observation, bracing and surgery. The Scoliosis Research Society (SRS) views physical therapy as an alternative treatment for scoliosis, along with yoga and chiropractic (among others) [1]. The official position of the SRS is that these methods have not demonstrated any scientific value and should not be used to formally treat the curvature, but can be utilized if they provide physical benefit. Increasing the level of evidence could influence physician attitudes on these treatment methods [2].

Chiropractic is a healthcare discipline that focuses upon the diagnosis and treatment of problems that affect the alignment of the muscles and bones of the body. Doctors of chiropractic are often perceived as doctors of the spine. This drives many scoliosis patients to self-select chiropractic care for their scoliosis, either as an alternative to bracing or surgery, or as a complementary or preventative measure; however, one study found that the procedures employed by most chiropractors to treat scoliosis are ineffective even in mild cases [3].

The chiropractic treatment of scoliosis is controversial. Chiropractic is not currently recognized as an accepted treatment for scoliosis in published guidelines [4]. Despite this, nearly 3 million scoliosis patients in the United States self-select chiropractic care for their condition every year [5]. This raises concerns among many healthcare professionals, as the lack of high-quality evidence renders drawing accurate conclusions regarding the chiropractic care of scoliosis difficult.

A scoliosis-specific chiropractic protocol was developed [6] that case reports [7–9] suggest may have greater potential to help scoliosis patients than general manual therapy [3, 10], possibly in the same way PSSE’s are preferable to general physiotherapy [11]. Due to concerns regarding the safety of chiropractic scoliosis treatment, an initiative was undertaken to identify potential side effects of scoliosis-specific chiropractic treatment and to lay the foundation for future quantitative research. This study aims to provide preliminary data regarding the frequency and severity of side effects reported immediately following scoliosis-specific chiropractic treatment, and to provide patients with the opportunity to document their perceived changes in health status immediately after treatment to determine how many patients felt worse after care.

## Methods

This study used a cross-sectional survey design involving a practice-based research network. 36 private chiropractic clinics specializing in the treatment of scoliosis who were involved in a practice-based research network and had completed *NIH Training for the Protection and Safety of Human Subjects in Research* were asked to participate. Of the clinics invited to participate, nine volunteered to contribute all relevant patient data over the calendar year of 2014. No compensation was provided to doctors or clinics for their participation.

Prior to the implementation of the study on January 1^st^, 2014, these nine clinics participated in a five-month trial period where various questionnaire designs were collected and evaluated for their ease-of-use and clarity. During this trial period, the patients became accustomed to filling out the forms properly after each visit, and the clinic staff were trained in how to collect the forms and handle them in such a manner that would not compromise patient confidentiality nor raise the possibility of patient acquiescence; namely, that patients would misrepresent their answers on the forms out of concern that they would be viewed by the treating chiropractor or their staff.

Standardized instructions were provided to the staff and to the patients prior to the beginning date of the study. The treating doctor was instructed not to view the forms and to ensure that patients knew that the forms would be kept confidential and would not be viewed by the doctor or their staff. Participating clinics were instructed to follow strict inclusion criteria and include only patients who were diagnosed with either adolescent or adult idiopathic scoliosis with Cobb angles measuring above 10 degrees accompanied by vertebral rotation (Lovett positive) and a positive Adam’s Forward Bending Test with no prior diagnoses of congenital or neuromuscular disorders nor prior surgical intervention.

Informed consent was obtained from every patient and patient participation was voluntary with no compensation. The collected response forms were sent to an independent researcher for analysis. The data was tabulated in a manner that removed all identifying information regarding the patients. To reduce any potential biases, the clinics were blinded to the results, forms did not have any indication of the treating doctor, and individual results from each clinic were not recorded separately.

The survey was provided to each scoliosis patient at the end of their clinic visit, and consisted of two questions: “How do you feel after your treatment today?” and “Did you experience any of the following as a result of your treatment today?” Possible responses to the first question included: Better; Same; or, Worse. Possible responses to the second question included: Muscle Soreness; Joint Stiffness; Nausea; Dizziness; Headache; Neck pain; Back pain; Strain/sprain; Discomfort from adjustment; Discomfort from adjusting instrument; Fracture; Stroke; Disc herniation; Joint dislocation; Open wound; and, Other (with an open-ended response allowed). Patients were allowed to select multiple side effects, but instructed to select only one answer to the first question. Side effects were broken down into three categories (mild, moderate, or severe). Sprains/strains were classified as moderate; fractures, strokes, disc herniations, joint dislocations, and open wounds were classified as severe. All other side effects were considered mild. Each visit was treated as a unique event.

### Description of the treatment protocol

The treatment applied to the patients in this study consisted of a standardized protocol that did not differ significantly at any of the participating clinics. This protocol included active spinal mobility exercises (such as side-bends), stretches, gentle axial traction and passive spinal distraction (dynamic loading and unloading of spinal discs and soft tissues), vibration therapy, massage therapy, chiropractic manipulative therapy (CMT), Whole-Body Vibration (WBV) therapy, and sensorimotor re-integration strategies (balance exercises performed with the aid of body weights and cantilevers on an unstable surface).

Active spinal mobility exercises, side-bends, and stretches were performed on a pivoting seat cushion for approximately 5 min. Intermittent over-the-door cervical axial traction was performed 100 times in two-second intervals over a time span of approximately 5 min. Passive spinal distraction therapy (sometimes also referred to as flexion-distraction therapy) was performed for approximately 15 min; this is a chiropractic technique primarily used for addressing intervertebral disc pathology and radiculopathies [12]. In this specialized manifestation, it is combined with a three-point stabilization system of straps to induce a “mirror-image” configuration of the scoliotic spine and used to address vertebral wedging. Passive vibration therapy was used to relax the para-spinal soft tissues and intervertebral discs to create a spinal-lengthening effect [13]. Massage therapy was performed, both manually and with the aid of a percussive massage device. CMT was provided to the thoracic and lumbar spine, and to the cervical spine with the aid of a mechanical precision adjusting instrument. Balance training and sensorimotor re-integration strategies were performed while the patient is standing upon a compliant surface placed on top of a Whole-Body Vibration (WBV) platform. This was accompanied by weights and cantilevers strategically placed on the patient’s body to create a reactive change in their posture. A form of WBV therapy was employed in a seated position, in conjunction with de-rotation, axial traction, and lateral traction.

## Results

From the nine participating clinics over the 2014 calendar year, 189 informed consent forms were collected (109 adolescents and 80 adults) and 3252 response forms were collected (1987 from adolescents and 1265 from adults), suggesting an average of 17 visits per patient. No patients declined to participate or discontinued participation midway through the study; however, 54 forms were discarded due to patients providing multiple answers to the question, “How do you feel after your treatment today; Better, Same, or Worse,” leaving a total of 3198 forms to be analyzed (1943 from adolescents and 1255 from adults).

Patients reported feeling worse post-treatment after 5.0 % of the visits. Side effects were reported after 29.7 % of visits. The most common side-effect reported was muscle soreness (accounting for 35.2 % of all side effects). The next common most side-effects were neck pain (13.6 % of side effects), back pain (12.0 %), headache (10.6 %), stiffness (7.8 %), and discomfort from the adjusting instrument (7.1 %). The most severe side-effect reported was a sprain/strain, which occurred 6 times. There were no reported incidents of open wounds, fractures, strokes, or joint dislocations immediately after treatment (Table 1).

**Table 1:** Side effects reported after treatment visit

	# of occurrences in adults	% frequency with adults	# of occurrences in adolescents	% frequency with adolescents	% of total side effects
Muscle soreness	244	19.4 %	362	18.6 %	35.2 %
Neck pain	80	6.4 %	154	7.9 %	13.6 %
Back pain	78	6.2 %	129	6.6 %	12.0 %
Headache	31	2.5 %	151	7.8 %	10.6 %
Stiffness	60	4.8 %	75	3.9 %	7.8 %
Discomfort from adjusting instrument	42	3.3 %	81	4.2 %	7.1 %
Discomfort from adjustments/ therapies	27	2.2 %	49	2.5 %	4.4 %
Dizziness	59	4.7 %	15	0.8 %	4.3 %
Nausea	16	1.3 %	15	0.8 %	1.8 %
Fatigue	6	0.5 %	18	0.9 %	1.4 %
Hip pain	7	0.6 %	5	0.3 %	0.7 %
Sprain/strain	4	0.3 %	2	0.1 %	0.3 %
Rib pain	4	0.3 %	0	0.0 %	0.2 %
Shoulder pain	2	0.2 %	1	0.1 %	0.2 %
Knee pain	1	0.1 %	1	0.1 %	0.1 %
Foot pain	2	0.2 %	0	0.0 %	0.1 %
Numbness/tingling	2	0.2 %	0	0.0 %	0.1 %

463 times (14.5 % of visits), the patient noted one or more side-effects as a result of treatment, but also reported feeling better. 331 times (10.4 % of visits), patients reported side-effects and stated they felt the same (Table 2).

**Table 2:** Patient-reported status after treatment visit

	Adult				
	No side effects	% of adult visits with no side effects	With side effects	% of adult visits with side effects	Total
Better	576	45.9 %	188	15.0 %	764
Same	294	23.4 %	126	10.0 %	420
Worse	3	0.2 %	67	5.3 %	70
Total	873	69.6 %	381	30.4 %	1255
	Adolescent				
	No side effects	% of adolescent visits with no side effects	With side effects	% of adolescent visits with side effects	Total
Better	835	43.0 %	275	14.2 %	1110
Same	539	27.7 %	205	10.6 %	744
Worse	1	0.1 %	88	4.5 %	89
Total	1375	70.8 %	568	29.2 %	1943
	Total (adult and adolescent combined)				
	No side effects	% of total visits with no side effects	With side effects	% of total visits with side effects	Total
Better	1411	44.1 %	463	14.5 %	1874
Same	833	26.0 %	331	10.4 %	1164
Worse	4	0.1 %	155	4.8 %	159
Total	2248	70.3 %	949	29.7 %	3198

## Discussion

With the exception of bracing, the side effects of conservative treatment for scoliosis have not been thoroughly investigated. A recent literature review found very low quality evidence that quality of life is not affected by wearing a brace, and that quality of life, back pain, and psychological and cosmetic issues did not change in the long term; the authors recommended additional research on pulmonary disorders, disability, back pain, psychological and cosmetic issues, quality of life, and side effects to improve the quality of the available evidence [14]. While wearing a brace can be stressful, a team approach may be helpful in recovering from stress and improving compliance [15].

This is the first study conducted specifically on the side effects of chiropractic scoliosis treatment, although a similar study involving a practice-based research network was conducted on the safety of pediatric chiropractic care; in this study, chiropractors reported three adverse events per 5348 office visits involving the care of 577 children, and parents reported two adverse events from 1735 office visits involving the care of 239 children [16]. The applied scoliosis-specific chiropractic protocol includes therapies and modalities which are not typically utilized in general chiropractic practice; for this reason, a more specific investigation into the safety and side effects of the chiropractic treatment of scoliosis is useful.

Studies on the safety and potential side-effects of CMT generally agree that adverse events are benign and self-limiting, and serious adverse events are rare [17–19]. In regards to infants and children, there are no published reports of deaths associated with chiropractic care, and published cases of serious adverse events are similarly rare [20]. No serious adverse events have been reported in any of the clinical studies for both adults and pediatric patients undergoing CMT [16, 21–26].

The incidence of side effects in this study did not differ significantly between adolescents and adults, with the exception of headaches and dizziness. Headaches occurred more than three times as often in adolescents compared to adults (7.8 % of adolescent visits, compared to 2.5 % for adults); this result is unexpected, and the literature is sparse concerning the relationship between scoliosis and headaches; one study found a history of headaches in one-third of children with idiopathic scoliosis and Arnold Chiari malformation Type I [27]. It is possible there could be an association between cerebellar tonsillar ectopia, headaches, and scoliosis. Dizziness occurred in 4.7 % of adults, compared to just 0.8 % of adolescents; this could be related to cardiovascular issues found more commonly in adults than teenagers.

A systematic review in 2009 found the frequency of adverse events after chiropractic interventions varied between 33 % and 60.9 % [19], comparative to the side effects reported in this study at 29.7 %. The most common side effects of CMT are soreness, neck pain, and headaches; these were reported after 18.9 %, 7.3 %, and 5.7 % of visits in this study, respectively. The most severe side effects of CMT are cardiovascular accidents (CVA’s) such as bleeding around the spine and stroke, which are estimated to occur between 1.5 times out of every 10,000,000 visits up to 5 times/100,000 visits. These rare but serious side effects are typically reported after CMT of the cervical spine, performed manually, that involves rotation of the cranio-cervical junction [28]. This type of CMT was not applied to any of the patients in this study, and no incidents of stroke were reported.

In regards to therapies common to chiropractic offices, anecdotal evidence suggests over-the-door manual traction is safe [29], particularly when performed actively by the patient. There are no reported side effects in the literature. The incidence of side effects from massage therapy has been reported to be around 10 %, all minor and self-limiting [30]. There are no published studies specifically investigating the side effects of spinal distraction therapy; however, no adverse events have been reported in clinical trials [31, 32].

In regards to modalities unique to the described scoliosis-specific protocol, vibration therapy was applied to the spine at a frequency around 4–5 Hertz; this has a resonant effect upon the spine [33], and in a weight-bearing state, this effect can be detrimental [34]. No studies have been conducted on the effects of introducing this frequency in a relaxed, supine state.

The main risk of balance exercises is the possibility of a fall resulting in a fracture or soft tissue injury. Special care was taken to ensure that patients undergoing balance training exercises had adequate supervision and handholds to prevent this from occurring; one of the main risks of a fall is a fracture, and no fractures were reported throughout the duration of this study. The risks to WBV exposure in an occupational setting are well-documented; [35] however, when used therapeutically, WBV appears quite safe, with no serious adverse events reported [36]. The amplitude, frequency, and type of vibration employed in this study were selected as to minimize these risks and to stay within the safe guidelines for WBV exposure as established by the International Standards Organization ISO-2631 [37]. The same considerations for the safety of seated WBV therapy apply as with the standing WBV therapy performed with balance training, minus the potential for falls. Stitzel et al. documented that the combination of seated vibration, axial traction, lateral traction, and de-rotation can be detrimental when improperly applied to the spine over extended periods of time [38]. In the same way that in-brace x-rays can be used to validate the corrective effect of a brace, in-chair x-rays can be used to ensure proper patient positioning to prevent this [39]. Commonly observed side effects of WBV therapy include nausea and dizziness; these were reported a combination of 106 times, or after 3.3 % of visits.

The most common side effect reported in this study was muscle soreness, accounting for over one-third of all side-effects. Although categorized as a mild side effect, muscle soreness could also be considered an inevitable consequence of an effective muscle rehabilitation and strengthening program. Future research on the side effects of PSSE’s could be helpful in this regard to determine if various physiotherapeutic scoliosis exercise programs result in a comparative incidence of muscle soreness and thus validate this hypothesis.

This study is a cross-sectional survey documenting the responses of scoliosis patients immediately after chiropractic treatment. As such, it has several limitations. Patient curve type, skeletal maturity indicators, and Cobb angles were not recorded; it is not known if certain subgroups would report a higher or lesser rate of side effects than others. The response forms were collected immediately after treatment and each treatment visit was treated as a non-longitudinal, unique event; therefore, conclusions cannot be drawn regarding the length or duration of the observed changes in health status or side effects. It is also unknown if some side effects would not be apparent immediately after treatment yet potentially manifest in the long-term. The purpose of the study was to determine if the chiropractic treatment of scoliosis was associated with an excessive frequency of severe side effects or negative changes in health status immediately following treatment; it was not intended to evaluate the effectiveness of the protocol. No objective outcome measures were included with the data collection for this study, and although the majority of patients reported feeling better immediately after treatment, it cannot be concluded that patient-reported changes in health status necessarily correlate with any objective assessments. For these reasons, no conclusions can be drawn regarding the effectiveness of the described intervention, either in the long-term or the short-term. Investigating the long-term effect of these therapies, while monitoring the patients for signs of adverse events, could be the goal of further research.

## Conclusion

Mild side effects were common, although the frequency was lower than the reported average for chiropractic interventions. The rate of moderate side effects reported was one per 533 visits involving the care of 189 scoliosis patients surveyed from 9 chiropractic offices over a timeframe of one calendar year. No serious adverse events occurred that required medical attention, hospital stays, or surgical intervention. Based upon this preliminary data, side effects reported by adult and adolescent idiopathic scoliosis patients immediately after chiropractic treatment appear to be relatively common but generally benign.
